# Drug repurposing improves disease targeting 11-fold and can be augmented by network module targeting, applied to COVID-19

**DOI:** 10.1038/s41598-021-99721-y

**Published:** 2021-10-19

**Authors:** Inés Rivero-García, Miguel Castresana-Aguirre, Luca Guglielmo, Dimitri Guala, Erik L. L. Sonnhammer

**Affiliations:** Department of Biochemistry and Biophysics, Stockholm University, Science for Life Laboratory, Box 1031, 17121 Solna, Sweden

**Keywords:** Computational biology and bioinformatics, Drug discovery

## Abstract

This analysis presents a systematic evaluation of the extent of therapeutic opportunities that can be obtained from drug repurposing by connecting drug targets with disease genes. When using FDA-approved indications as a reference level we found that drug repurposing can offer an average of an 11-fold increase in disease coverage, with the maximum number of diseases covered per drug being increased from 134 to 167 after extending the drug targets with their high confidence first neighbors. Additionally, by network analysis to connect drugs to disease modules we found that drugs on average target 4 disease modules, yet the similarity between disease modules targeted by the same drug is generally low and the maximum number of disease modules targeted per drug increases from 158 to 229 when drug targets are neighbor-extended. Moreover, our results highlight that drug repurposing is more dependent on target proteins being shared between diseases than on polypharmacological properties of drugs. We apply our drug repurposing and network module analysis to COVID-19 and show that Fostamatinib is the drug with the highest module coverage.

## Introduction

Drug discovery has traditionally been centered around the “one drug—one gene—one disease” paradigm with the aim of achieving a therapeutic outcome while minimizing detrimental off-target effects. This perspective has proven to be successful in some cases, like the BCR-ABL tyrosine kinase inhibitor Imatinib^1^, but solely adhering to this model has its downsides. Firstly, the drug development process requires large investments to be successful, averaging at $2–3 billion and 13–15 years per medication to achieve regulatory marketing approval^2^. Secondly, and as a consequence of the financial risks, not all diseases are targeted in drug discovery, which leaves patients affected by rare conditions with limited therapeutic options^3^.

Two concepts that could mitigate these problems are polypharmacology and drug repurposing. The concept of polypharmacology refers to the ability of some drugs to target more than one protein^4^. Although it might appear as undesirable at first, polypharmacology can modulate several cellular pathways simultaneously, thereby increasing treatment efficacy^5^^.^ Drug repurposing or repositioning, defined as the use of an approved drug for a new therapeutic indication^6^, can make the market life of a drug more appealing by decreasing development costs to $40–80 million and 3–12 years for a new indication^2^. The combination of polypharmacological and repositioning strategies could offer therapeutic opportunities for patients with any condition, and its importance is strongly exemplified in the case of fast-evolving pandemic diseases such as the ongoing COVID-19, which has to date caused more than 212 million cases and more than 4 million deaths^7^. Worldwide efforts to find therapeutic candidates for COVID-19^8–11^, such as the Coronavirus Treatment Acceleration Program by the FDA^12^, have been put into action and would benefit from therapeutic opportunities provided by drug repurposing in order to facilitate resource planning and allocation. We here approach drug repurposing from a network and module-based perspective and demonstrate its value for COVID-19.

Networks of functional associations present a convenient model of intracellular relations between proteins, including physical, regulatory, and functional interactions^13^. Such networks exhibit emergent properties^14^ on the systems level that manifest themselves in phenotypes and diseases^15^, that are not encoded in a single gene. Even for Mendelian diseases, where phenotypes are caused by single mutations, there is a plethora of modifier genes influencing the final outcome^16^. In general, genes associated with a given disease tend to cluster together when mapped to a functional association network, forming so-called disease modules^17^. For a disease phenotype to manifest itself, the integrity of the underlying disease module needs to be perturbed^18^. The disease module hypothesis has increased our understanding of molecular pathological mechanisms and has been successfully applied to improve therapeutic strategies^19^.

**Figure 1:**
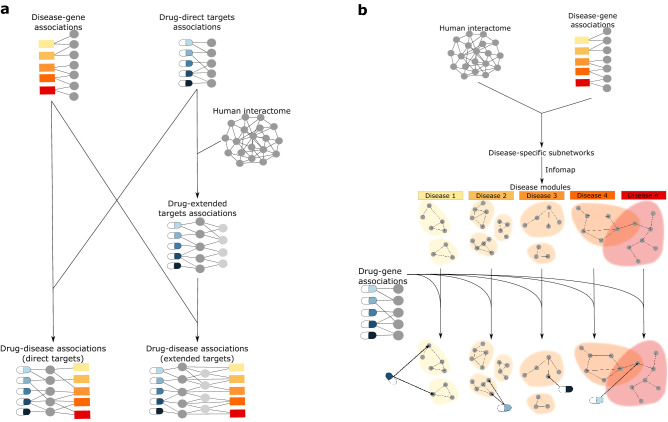
Schematic overview of study workflow. (**a**) The human interactome (FunCoup^38^) was used to map drug target associations for FDA-approved drugs from DrugBank^40^ and disease-gene associations from DisGeNET^42^. The sets of drug targets were also extended using first order neighbors in the human interactome. The overlap between drug targets and disease gene sets was analyzed to determine the potential disease leverage that could be offered by repurposing. (**b**) In order to find disease modules, the interactions between disease genes retrieved from the human interactome were partitioned using Infomap^47^. The overlap between drug targets and disease genes was later analyzed to map drugs to the disease modules.

Advantages, strategies, and successful implementations of network-based drug repurposing have been described previously^20–23^. However, the extent of the therapeutic opportunities that can be gained from drug repurposing has to our knowledge not yet been assessed. In this study we combine drug targets with disease-associated genes to determine the extent of therapeutic opportunities to be gained from network-based drug repurposing, both with and without using a network (Fig. 1a). Additionally, the analysis is performed in the context of disease modules found in the human functional interactome (Fig. 1b). Our main findings suggest that drug repurposing can offer an average 11-fold potential increase in drug–disease associations, here referred to as disease leverage, and that polypharmacological drugs tend to have all targets in one or a few modules. These findings lead us to conclude that drug repurposing at the module level could benefit more from the pleiotropic nature of some disease genes than from the polypharmacological action of drugs.

## Results

To gain insights into the therapeutic opportunities yet to be exploited from drug repurposing we performed three levels of analyses: at the drug target level, at the disease level and at the disease module level (Fig. 1). With respect to the target level, we quantified the number of direct targets per drug and also extended these to consider their first neighbors in the human functional interactome. Regarding the disease level, we quantified the number of diseases each drug can be linked to by mapping drug targets to diseases. Using the same method, we also analyzed how drugs are linked to disease modules.

### Direct drug–protein target associations: most drugs target few proteins.

**Figure 2:**
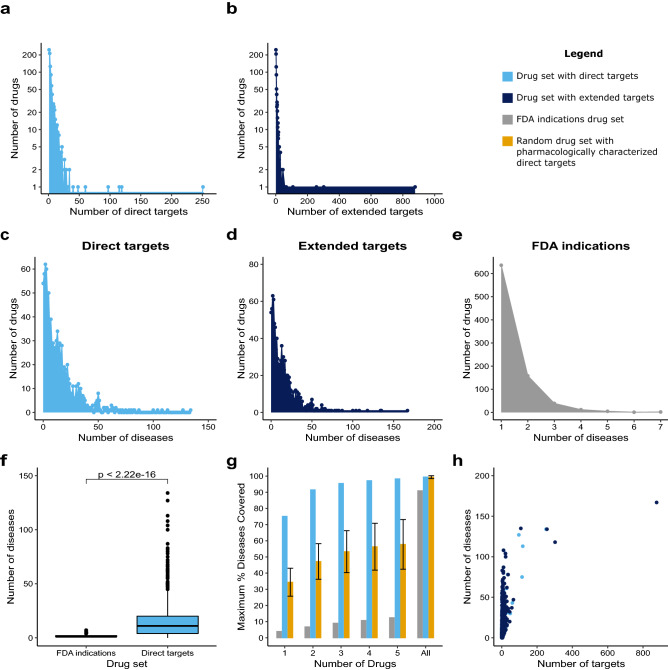
Drug repurposing may offer an average 11-fold increase of disease leverage. (**a**) Distribution of the number of genes targeted by FDA-approved drugs e.g., drug targets. (**b**) Distribution of the number of extended drug targets. (**c**) Distribution of the number of diseases covered by direct targets of FDA-approved drugs. (**d**) Distribution of the number of diseases covered by the extended targets of the drug set. (**e**) Distribution of the number of diseases targeted per drug for the “FDA indications” data set. (**f**) Statistical comparison of the distributions of disease coverage between the “FDA indications” data set and the direct drug target data set. (**g**) Quantification of the maximum number of diseases covered by 1, 2, 3, 4, 5 and all drugs for the “FDA indications”, the drug set with direct targets and a randomized version of the drug set. In the randomized drug set each drug has the same number of targets as in the original drug set, but the target genes are randomly chosen from the human genome. This random drug set represents the background levels of disease coverage. (**h**) Correlation between the number of targets and the number of diseases mapped to a drug. For the direct targets: rho = 0.636, p-value < 2.2 × 10^–16^. For the extended targets: rho = 0.644, p-value < 2.2 × 10^–16^.

To establish the specificity of the currently approved drugs we assessed the number of proteins targeted by each of the drugs. The number of direct protein targets per drug ranges from 1 to 251, with a median value of 3 (Fig. 2a). After ranking the drugs according to the number of proteins they target we could identify the spleen tyrosine kinase (SYK) kinase inhibitor Fostamatinib, used e.g., in the treatment of rheumatoid arthritis and immune thrombocytopenia purpura^24^, at the top of the list. We note that 6 of the 10 top drugs in this ranking are naturally occurring ions and small molecules that act as enzyme cofactors, such as NADH or Copper (Sup. Table 1). When only the pharmacologically characterized targets of a drug were considered (e.g., proteins for which the action of the drug on them has been experimentally characterized) the distribution of direct targets per drug has a similar shape, with the number of direct targets ranging from 1 to 33 and a median value of 1 (Sup. Fig. 1 online). The top-ranked drug is NADH, a nutraceutical drug used e.g., in the management of Parkinson's disease and in dietary supplementation therapies^25^. Also among the top ranking drugs are the GABA-receptor inhibitors Clotiazepam, Clonazepam, and Flurazepam (Sup. Table 2 online). The top-10 drugs in these two rankings are very different, and it is worth noting for pharmacologically characterized targets that there is only one naturally occurring molecule in humans, NADH, while the “all targets'' top-10 list contains 6 such drugs. This highlights the target specificity principle that guides drug design and facilitates marketing approval.

### Extending the target sets: drug repurposing perspectives considering network neighbors

Because the pharmacological perturbation of a protein can affect its interacting partners, we also extended the set of direct targets with their network neighbors and performed the same analysis. After target extension, drugs had between 1 and 877 extended targets, with a median of 3 (Fig. 2b). When only the pharmacologically characterized direct targets were considered, the distribution of number of extended targets per drug ranged from 1 to 172 with a median of 1 (Sup. Fig. 1 online). In both cases, the ranking of the top scoring drugs is highly similar to the ranking produced by studying only the direct targets. Some exceptions exist—for example cholic acid, used for the treatment of e.g. peroxisomal and bile acid synthesis disorders, climbed from position 7 in the direct targets ranking to position 9 in the extended targets ranking (Sup. Table 1 online, respectively).

### Drug–disease associations: an average 11-fold disease leverage from drug repurposing

To assess the distribution of FDA-approved drug–disease associations, the “FDA indications” dataset was used. The distribution of FDA indications per drug ranged from 1 to 7, with a median value of 1 (Fig. 2e). It is noteworthy that 6 out of the top 10 drugs associated with the most diseases (Sup. Table 3 online) are biotechnological products, e.g., pharmacological macromolecules derived from or produced in biological systems such as tissues or cells. Examples include the soluble TNF receptor recombinant protein Etanercept, used in the treatment of rheumatoid arthritis^26^, and the immune checkpoint inhibitor antibody Avelumab, which prevents the PD1/PDL1 interaction that restricts the immune defense against tumors^27^.

An overlap between a drug´s targets and genes associated with a certain disease could indicate a potential therapeutic effect of the drug on the disease phenotype. To estimate how many diseases are targeted by each drug, we systematically studied this overlap in the “direct targets” dataset. The overlap ranged between 0 and 134 diseases, with a median of 11 diseases, per drug (Fig. 2c). The drugs targeting more proteins tend to show an overlap with a larger number of diseases (Fig. 2h). Fostamatinib was once again ranked at the top of this list, together with several drugs with anti-inflammatory properties such as Aspirin, Ibuprofen and Dexibuprofen (Sup. Table 1 online). As drugs with FDA indications only target 1 disease on average, this suggests that drug repurposing on average offers an 11-fold increase in disease coverage (Fig. 2f). If only the pharmacologically characterized drug targets are considered, each drug targets between 0 and 106 diseases (Sup. Fig. 1 online, Sup. Table 2 online). This still increases the average disease leverage fivefold as compared to the FDA indication drug–disease associations (Sup. Fig. 1 online). For both all targets and pharmacologically characterized targets only, the drug targets are four times more shared between diseases than would be expected (Sup. Fig. 2 online, p-value < 2.2 × 10^–16^).

### Combinatorial drug therapy

To further examine the opportunities that drug repurposing opens for disease management, we calculated the maximum number of diseases that a small number of drugs can cover (Fig. 2g). Strikingly, already three drugs can cover 95% of all diseases. These drugs are Fostamatinib, Zinc, and Neonatal foreskin keratinocyte. We note that the disease coverage is dramatically higher than for FDA indications, which only achieves maximally 10% of the diseases for 5 drugs. The disease coverage up to 5 drugs is significantly (p-value < 2.2 × 10^–16^) higher than expected by random sampling (Fig. 2f). Similar results were obtained when considering the pharmacologically characterized drug targets only (Sup. Fig. 1 online). These results support the fact that drug repurposing has a high potential for combinatorial therapeutic opportunities yet to be exploited.

### Increased disease coverage after extending the drug targets

To study the effects of extending the sets of drug targeted proteins on the coverage of the diseasome we assessed the overlap of the extended drug target sets and disease genes. Together, all drugs in the data set cover 176 of the 177 diseases. Individually, drugs can cover between 0 and 167 diseases, with a median of 11 diseases (Fig. 2d, Sup. Table 1 online). If only the pharmacologically characterized targets are extended the number of diseases covered by a single drug ranges between 0 and 106, with a median of 5 (Sup. Fig. 1 online, Sup. Table 2 online). This suggests that modulating a protein by the pharmacological perturbation of one of its functional neighbors could give further opportunities for disease management by drug repurposing.

### Drug–disease module associations: opportunities for drug repurposing at the disease module level

Based on the hypothesis that the perturbation of disease modules is the underlying cause of disease, we sought to examine how drugs target disease modules. The community finder Infomap retrieved disease modules of at least three genes for 157 out of the 177 diseases in the data set (89%). The median number of modules per disease was 2 with a range from 1 to 26. Experimental liver cirrhosis demonstrated the highest number of modules. The identified disease modules had a median size of five genes. The largest disease module was found for COVID-19, where 423 of 572 genes were part of a single connected component (Sup. Fig. 3 online).

**Figure 3:**
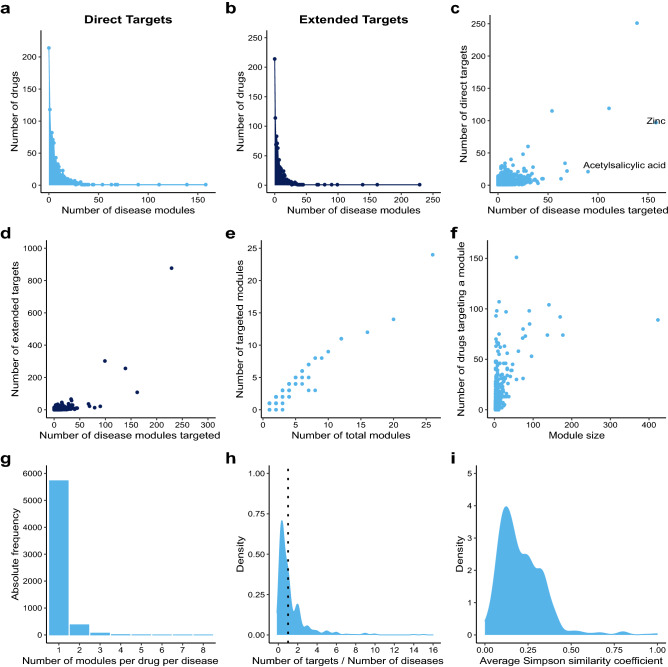
Most drugs target few modules. (**a**) Distribution of the number of modules targeted per drug considering the direct targets. (**b**) Distribution of the number of modules targeted per drug considering their extended targets. (**c**) Correlation between the number of disease modules targeted by a drug and its number of direct gene targets (rho = 0.495, p-value < 2.2 × 10^–16^). (**d**) Correlation between the number of disease modules targeted by a drug and its number of extended gene targets (rho = 0.503, p-value < 2.2 × 10^–16^). (**e**) Correlation between the total number of modules and the number of drug-targeted modules in a disease (rho = 0.903, p-value < 2.2 × 10^–16^). (**f**) Correlation between the size of a disease module (number of genes) and the number of drugs targeting it (rho = 0.488, p-value < 2.2 × 10^–16^). (**g**) Number of modules targeted per drug in a disease targeted by that drug. (**h**) Density plot of the number of direct targets per disease for all drugs (average ratio = 0.52). (**i**) Density plot of the Szymkiewicz–Simpson similarity coefficients between disease modules targeted by single drugs (average Szymkiewicz–Simpson similarity coefficient = 0.211).

To find the most universal drugs at the module level, we counted the number of modules targeted by each drug on a gene-overlap and disease-independent basis. This number ranged from 0 to 158 (32%) modules, with a median value of 4 (Fig. 3a). Overall, the top drugs in this ranking were highly similar to the best ranked ones based on diseases, with Zinc as the top-ranked drug (Sup. Table 1 online). The similarity may be explained by the fact that the number of diseases and disease modules covered by single drugs is highly correlated (Pearson’s correlation coefficient = 0.98). When only the pharmacologically characterized drug targets are considered the distribution ranged between 0 and 44, with a median of 1 and Neonatal foreskin keratinocyte as its best performing agent (Sup. Fig. 4 online, Sup. Table 2 online).

We also investigated how the extension of the drug targets affected drug targeting at the disease module level. Single drugs with extended targets covered between 0 and 229 disease modules (46%), with a median value of 4 (Fig. 3b). The best performing drug in this category was Copper (Sup. Table 1 online). For pharmacologically characterized targets, between 0 and 74 modules were targeted per drug with a median of 1. Fostamatinib was then the highest ranked drug (Sup. Fig. 4 and Sup. Table 2 online). The high correlation between the number of diseases and the number of modules targeted by single drugs was maintained when using the extended targets (Pearson’s correlation coefficients = 0.98).

Given that most drugs target few modules, are the targets of a drug typically in the same module? To address this question, we calculated the correlation between the number of direct targets of a given drug and the number of modules it targets. If the targets of a drug were present in different modules we could expect a high positive correlation between these two variables. However, the Spearman correlation is only 0.495, thus targets of one drug tend to be part of the same disease module, albeit with some outliers such as Acetylsalicylic acid, which targets 90 disease modules with just 21 targets (Fig. 3c). Similar results were obtained for the extended drug target sets (Fig. 3d).

**Figure 4:**
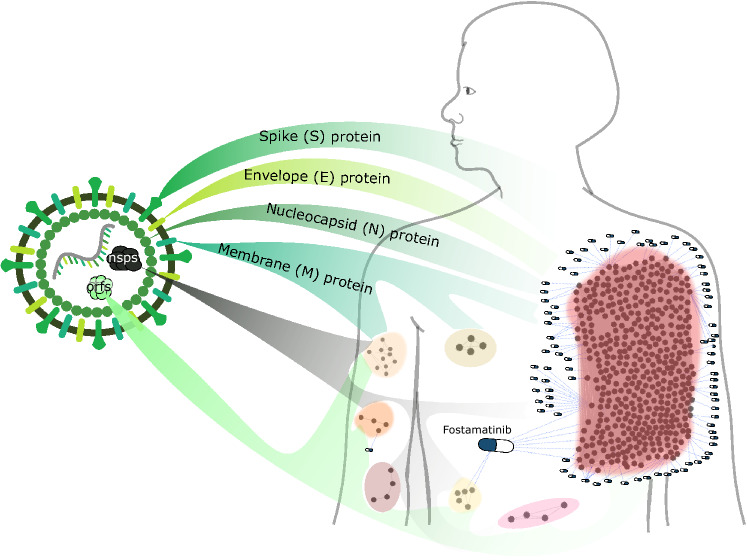
Drug targeting of the COVID-19 disease modules. Bipartite network linking drugs with their targets in the COVID-19 disease modules, where nsps are the SARS-CoV-2 non-structural proteins and orfs are its other open reading frames. Several polypharmacological drugs can be observed, highlighting Fostamatinib as the only one that is linked to two modules. The remaining polypharmacological drugs are linked to one module only.

As an example of module targeting, Fig. 4 shows a bipartite network of drugs and disease genes in COVID-19. This network is composed by the human proteins that interact with SARS-CoV-2 proteins^28^ and the 90 drugs (Sup. Table 4 online) that target them. We found seven modules in the COVID-19 network. Although there are several polypharmacological drugs targeting this disease, Fostamatinib is the only one that targets different modules. Fostamatinib has been found to reduce the levels of membrane-bound MUC1^29,30^, the levels of Neutrophil Extracellular Traps^31^ and platelet activation^32^. Altogether, this results points toward the potential beneficial effect of Fostamatinib in severe COVID-19 patients, which is being currently evaluated by two clinical trials (Clinicaltrials.gov identifiers: NCT04579393 and NCT04581954^33^). Moreover, the imbalance in the number of drugs targeting the different modules points towards the importance of employing drugs that together can target all modules (e.g., pathophysiological mechanisms) involved in the disease.

With the hypothesis that different modules represent different pathobiological molecular mechanisms, we investigated if diseases with a higher number of modules have more modules that are targeted by drugs. The correlation between the total number of modules and the number of drug-targeted modules in a given disease supports this idea (Spearman correlation coefficient = 0.90, Fig. 3e). Since there is also strong correlation between the number of genes associated with a disease and its number of modules (Spearman’s correlation coefficient = 0.77, Pearson’s correlation coefficient = 0.89), a disease with more genes has higher chances of having more modules as well as a higher chance of being targeted by a drug.

**Figure 5:**
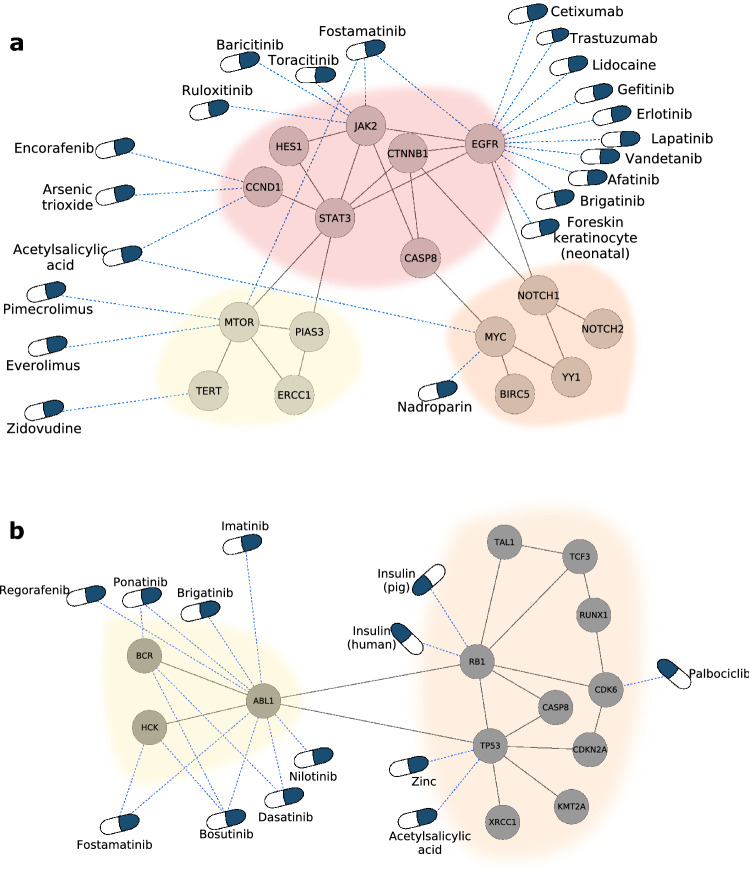
Network examples of disease module targeting by drugs. (**a**) Disease modules for uterine cervical neoplasm accompanied by the drugs that target them. The biggest module is the one associated with most drugs. There are two polypharmacological drugs: Acetylsalicylic acid and Fostamatinib, both of them targeting two out of the three disease modules. (**b**) Disease modules for childhood acute lymphoblastic leukemia with the drugs that target them. In this case the smallest module is the one associated with most drugs. There are four polypharmacological drugs (Ponatinib, Fostamatinib, Bosutinib and Dasatinib), all of them associated only with the small module.

Is the number of drugs targeting a module affected by the size of the module? The correlation between these two variables was found to be 0.535 (Fig. 3f), suggesting a general trend, although there are several diseases in which the smallest module has the highest number of drugs. As an example of the general trend, 76% (19 of 25) of the drugs targeting uterine cervical neoplasm target the largest module of 7 genes, while the rest of the drugs are divided between two modules of 5 and 4 genes respectively (Fig. 5a). An example of the opposite trend is childhood acute lymphoblastic leukemia, where more than 60% (8 of 13) of the drugs target the smallest module of 3 genes, while the largest module with 10 genes is targeted by only 5 drugs (Fig. 5b). For drugs targeting a single disease, the most common pattern is that each drug targets a single module, yet exceptions exist where up to 8 modules are targeted by a single drug, which is the case for Fostamatinib in experimental liver cirrhosis and malignant neoplasm of breast (Fig. 3g). Further examples can be found in Sup. Fig. 5 online.

Lastly, we asked whether drugs targeting more than one disease do this by targeting the same or distinct proteins in different diseases. Our results show that on average, drugs have 0.75 targets per disease (Fig. 3h), e.g., there is a clear trend that multiple diseases are targeted because they share the same target. We further calculated the overlap between disease modules targeted by a single drug. The distribution of these Szymkiewicz–Simpson coefficients shows that the modules targeted by the same drug are very different from each other (Fig. 3i). Putting these results together, we conclude that drug repurposing opportunities are mainly due to the pleiotropic nature of disease genes, rather than polypharmacological properties of drugs.

## Discussion

The main aim of this study is to assess the disease leverage that can be achieved by drug repurposing, either via direct targeting or via a network of functional interactions. We also studied how network modules within disease gene sets are targeted. Our findings demonstrate that drug repurposing can offer an 11-fold increase in disease coverage on average, and that disease modules can be used to pinpoint untargeted pathological mechanisms and identify polypharmacological drugs that can perturb a module at multiple targets, reducing the chances of drug resistance^34^.

At first, we established some basic characteristics of approved drugs. The low number of direct targets per drug, median value of 3, is in line with the traditional aim of drug discovery^6^ to minimize off target effects making the mode of action more easily explainable and avoiding adverse drug reactions. After having examined direct drug targets, we sought to quantify disease coverage by available drugs to answer our main research question: what is the potential of drug repurposing with respect to disease coverage. Our results show that drug repurposing can offer an average 11-fold increase in disease leverage, giving significant results when compared to a background set of target genes. While most of this repurposability potential is explained by gene pleiotropy, there is an average twofold increase in disease leverage from polypharmacological properties of drugs alone. Moreover, expanding the sets of direct drug targets prior to repurposing may increase disease coverage even further and could provide novel therapeutic strategies.

In an attempt to facilitate identification of drug repurposing candidates, using the disease module hypothesis, we have generated a table that maps each disease gene to disease(s), module(s), and drug(s), either directly or via network extension (Sup. Table 5 online). Pharmacological characterization is also indicated. This resource could be used to identify repurposing candidates given a disease or a set of genes. Moreover, it can be used to find additional drugs that target other modules, given an existing drug–disease combination. However, additional studies should be performed to assess if a given drug can be repurposed for a particular disease because the fact that a drug has targets in a set of disease genes does not guarantee a therapeutic effect on that disease. Gene expression signature studies and structural predictions could help to assess drug–disease compatibility and to select the most suitable candidates for further testing^35^.

Genes associated with a single disease typically do not form a single connected network component. This could be due to the incompleteness of the network^18^ but also to the existence of multiple mechanisms, each linked to a distinct subset of genes forming distinct disease modules. Methods such as Disease module detection (DIAMOND)^36^ and Seed connector algorithm (SCA)^37^ aim to connect the genes of a disease into larger modules by adding connector genes. In the case of DIAMOND, submodules are then detected by clustering methods. We here followed a similar approach that allows disease genes to be part of multiple distinct modules. This idea is supported by the facts that diseases are often heterogeneous and that disease genes can be associated to either the cause or the effect of the disease. To define disease modules we used InfoMap which is a widely used module detection algorithm that has performed well in benchmarks^38^.

The identification of disease modules was done in a disease-independent manner, which resulted in low similarity between modules of different diseases (average Szymkiewicz–Simpson similarity coefficient = 0.015). We also found that drugs tend to target very few disease modules, which once again points towards a very targeted approach in drug design. Moreover, drug targets tend to be part of the same disease module, and diseases with more modules tend to be targeted by more drugs, despite lack of a clear correlation between module size and the number of drugs that target it. The asymmetry of drug targeting across modules opens the possibility of finding drugs with the widest coverage at the disease module level. Such drugs, like Fostamatinib in the COVID-19 case, can potentially achieve higher efficacy because they can interfere with more mechanisms.

This study explores and showcases the therapeutic potential that could be obtained from drug repurposing in conjunction with network analysis. Disease modules can be helpful for GWAS interpretation^39^, identifying key pathological proteins^40^, guiding the design of effective therapeutic strategies^41^, and detecting drug repurposing opportunities^42^. These strategies could increase treatment efficacy by targeting multiple disease modules and genes, and selecting the drug targets that lead to a therapeutic, rather than symptomatic, treatment of disease^3^. Looking forward, the use of the human interactome information for drug repurposing strategies may lead to economic, social and medical benefits in the treatment of human disease.

## Methods

### Human interactome

The human interactome was retrieved from FunCoup v4.1^43^. FunCoup is an online resource of functional association networks in multiple species. It is constructed by naïve Bayesian integration of ten different types of evidence of functional interactions, including: domain–domain and protein–protein interactions, mRNA and protein co-expression, genetic interactions, co-regulation by transcription factors and micro RNA and co-evolution. Additionally, the functional associations are transferred to multiple species using orthologs identified by InParanoid^44^. The retrieved *Homo sapiens* interactome from FunCoup contains 5,315,787 interactions among 17,402 nodes and was used because it is the largest network comprised exclusively of experimental data. The human interactome data was used to expand the drug target gene sets and identify interactions between disease genes needed for identification of disease modules.

### Drug–gene targets data set

Drug–gene target data was retrieved from DrugBank (version 5.1.5)^45^. To ensure the clinical utility of the results, only FDA-approved drugs were considered. Subsequently, drug–gene mappings for which a gene lacked an Ensembl ID or was not present in FunCoup were removed. Drugs with identical gene targets and indications were grouped together keeping only one drug as a group representative while removing the rest of the group from the analysis. This yielded a drug set consisting of 985 drugs. Additionally, the Therapeutic Target Database, TTD (2020 version)^46^, was used to subset the drug–target gene collection, keeping only experimentally characterized drug–gene mappings e.g., those for which an exact pharmacological action of the drug on the gene has been experimentally determined. This data set was referred to as “pharmacologically characterized targets” and consists of 762 drugs. These two drug sets were expanded to include the first neighbors of the drug targets, from FunCoup with a link confidence score pfc ≥ 0.99. An additional drug set that mapped each drug with its FDA-approved indications was obtained from TTD^41^ and named “FDA indications”. Table 1 summarizes the sizes of all drug data sets.

**Table 1:** Drug–gene targets data sets. FDA indications only connect drugs to diseases, not to targets.

Data set	Number of drugs	Number of genes	Number of drug–gene mappings
Drugs with direct targets	985	1590	5286
Drugs with pharmacologically characterized direct targets	762	527	1374
Drugs with extended targets	985	6017	1374
Drugs with pharmacologically characterized extended targets	762	4119	44,386
FDA indications	853	NA	NA

### Disease genes data set

Disease genes were retrieved from DisGeNET v6.0^47^. Only the disease-gene associations reported by the Comparative Toxicogenomics Database, CTD^48^, were kept, as those have been manually curated by the authors of CTD. Conditions classified as phenotypes or disease groups were removed. Diseases with fewer than 20 genes were removed in order to keep only well-characterized diseases, as in previous work^17^. Lastly, to correct for the fact that diseases do not have an unambiguous nomenclature in DisGeNET, e.g., cerebral artery atherosclerosis and cerebral atherosclerosis, the remaining diseases were merged under the same name if their Szymkiewicz–Simpson similarity coefficient^49^, calculated as shown in Eq. (1), was equal or greater than 0.95. This threshold was selected because it maximizes the number of disease-gene associations while minimizes the overlap between diseases.

1 $$ overlap\left( {X, Y} \right) = \frac{{\left| {X \cap Y} \right|}}{{{\text{min}}\left( {\left| X \right|, \left| Y \right|} \right)}} $$

In addition, the 572 human genes associated with COVID-19 reported by Gordon et al*.*^50^ and Li et al*.*^51^ were retrieved from IntAct^28^ and added to the dataset. The final data set contained 13,560 disease-gene associations between 177 diseases and 5766 genes.

### Direct and extended target-based drug ranking

For the direct target ranking, drugs were sorted by the number of direct gene targets. In the extended target ranking, direct drug targets were expanded using their first order neighbors retrieved from FunCoup with confidence pfc ≥ 0.99. From these, high quality first neighbors were retrieved using MaxLink^52^. Maxlink is a guilt-by-association algorithm that identifies genes tightly linked to a set of query genes using a hypergeometric test to ensure the statistical significance of the association. MaxLink was run independently for each drug, with the drug direct targets and the gene interactions with pfc ≥ 0.99 in the human FunCoup v4.1^43^ network as inputs. The neighbor genes with an Benjamini–Hochberg FDR-corrected^53^ p-value ≤ 0.05 were returned. The drugs were then sorted by the number of expanded targets.

### Disease-based drug ranking

Drugs were mapped to a disease if one or more of its gene targets was part of the disease gene set. All drugs were then sorted based on the number of diseases they had been mapped to. A similar procedure was done for the extended drug targets. The drugs in the “FDA indications” data set were ranked based on number of indications.

### Disease coverage by drugs

To analyze how many diseases can be covered by n drugs (n = 1, …, 5 or the full data set) the direct targets drug set and the “FDA indications” drug set were reduced in an iterative procedure. In each iteration, the drug covering the highest number of diseases in terms of gene-overlap was removed, and the number of diseases was added to a counter. The covered diseases were removed from the diseases-to-target space e.g., diseases not yet covered during the iteration. The procedure was repeated n times. A permutation test was performed to assess the statistical significance of the disease coverage. The test involved performing the drug coverage estimation procedure with randomly sampled gene sets of the same size as the drug target sets, repeated 1000 times.

### Finding disease modules

For each disease in the disease data set, the interactions between disease genes were retrieved from FunCoup using the link confidence cutoff of pfc ≥ 0.80). Infomap^54^ was used to find modules in the subnetworks, using the pfc scores as edge weights. For each disease, one or more disease modules were retrieved. Subsequently, and following Choobdar et al.^55^, disease modules with fewer than three genes were removed. The final set contained 503 modules for 157 diseases. The set of disease modules and their corresponding genes is summarized in Sup. Table 6 online.

### Disease module-based drug ranking

Disease module-based drug rankings were built for the direct targets and extended targets drug sets. In both cases drugs were mapped to disease modules with which they had at least one overlapping gene. Then, the drugs were sorted based on the number of disease modules they overlap with.

### Disease module representations

Cytoscape v3.2.1^56^ and Inkscape v1.0.2 (https://inkscape.org/) were used to visualize disease modules and the bipartite drug–disease module networks.

## Supplementary Information

Supplementary Table 1.

Supplementary Table 2.

Supplementary Table 3.

Supplementary Table 4.

Supplementary Table 5.

Supplementary Table 6.

Supplementary Figures.

Dataset S1.

Dataset S2.

Dataset S3.

## Data Availability

All the data that support the findings of this study are available at https://bitbucket.org/sonnhammergroup/unadrug.

## Code availability

The codes used in this project are available at https://bitbucket.org/sonnhammergroup/unadrug.
