# Supplementary material for: Drug repurposing improves disease targeting 11-fold and can be augmented by network module targeting, applied to COVID-19
Source: Sci Rep. 2021 Oct 19;11:20687. doi: 10.1038/s41598-021-99721-y (PMC8526804; doi:10.1038/s41598-021-99721-y)
Supplement: Supplementary file 7 — Supplementary Figures. [file 41598_2021_99721_MOESM7_ESM.pdf]

Drug repurposing improves disease targeting 11-fold on average. New insights from network module targeting and its application to COVID-19.

Inés Rivero-García, Miguel Castresana-Aguirre, Luca Guglielmo, Dimitri Guala,
Erik L. L. Sonnhammer*

Department of Biochemistry and Biophysics, Stockholm University, Science
for Life Laboratory, Box 1031, 17121 Solna, Sweden,
*To whom correspondence should be addressed:
erik.sonnhammer@dbb.su.se

Supplementary figures

Supplementary figure 1

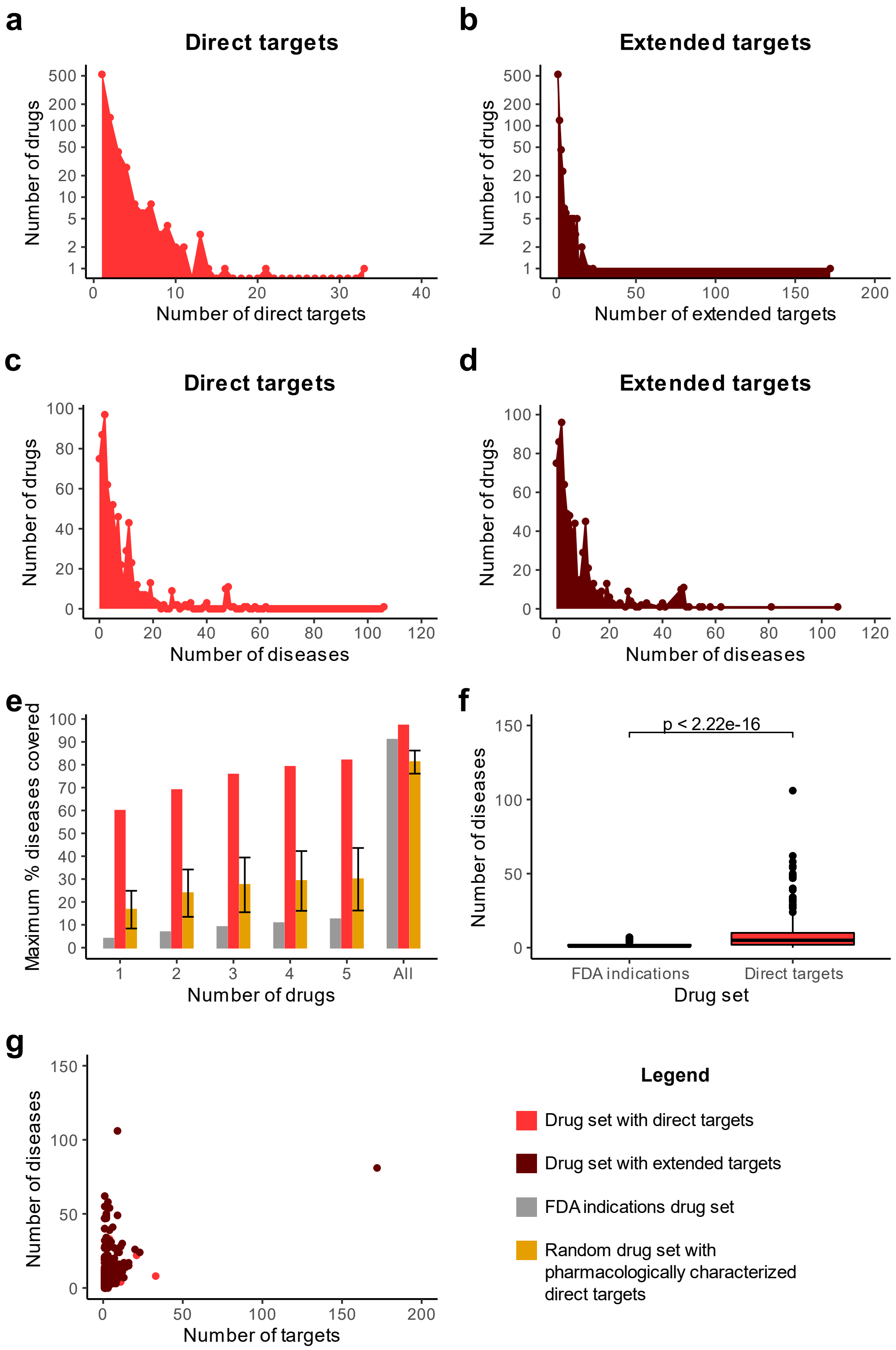

Drug repurposing using characterized pharmacological targets might offer an average 6-fold increase in disease leverage. a) Distribution of the number of pharmacologically characterized direct gene targets per drug. This number ranges from 1 to 33, with a median

of 1 and a standard deviation of 2.18. **b)** Distribution of the number of extended gene targets per drug when using the pharmacologically characterized targets as extension seeds. This number ranges from 1 to 172, with a median 1 and a standard deviation of 6.61. **c)** Distribution of the number of diseases covered by single drugs, considering their pharmacologically characterized direct targets only. The distribution ranges from 0 to 106, with a median of 5 and a standard deviation of 10.958. **d)** Distribution of the number of diseases covered by single drugs when their pharmacologically characterized direct targets are extended. The distribution ranges from 0 to 106, with a median of 5 and a standard deviation of 11.43**. e)** Quantification of the maximum number of diseases covered by 1, 2, 3, 4, 5 and all drugs of the "FDA indications" data sets and the drug set with pharmacologically characterized direct targets. This drug set covers 97.18% of diseases, a significantly higher number than then 90.96% of diseases covered by the "FDA indications" data set (p-value = 0.02). For low numbers of drugs, the number of diseases potentially covered by the pharmacologically characterized direct drug targets is also higher than the "FDA indications" one (p-values < 2.2×10^{-16} in all cases). Regarding the background levels, in all cases the proportion of diseases covered by the drug set with only pharmacologically characterized direct targets is significantly higher than the background levels (p-values of 9.17×10^{-16}, 7.04×10^{-16}, < 2.2×10^{-16}, < 2.2×10^{-16}, < 2.2×10^{-16} and 2×10^{-4}, respectively). **f)** Statistical summary of the distributions of panels C and Fig. 2E. **g)** Correlation between the number of targets and the number of diseases mapped to a drug. For the direct targets: rho = 0.372, p-value < 2.2×10^{-16}. For the extended targets: rho = 0.402, p-value < 2.2×10^{-16}).

Supplementary figure 2

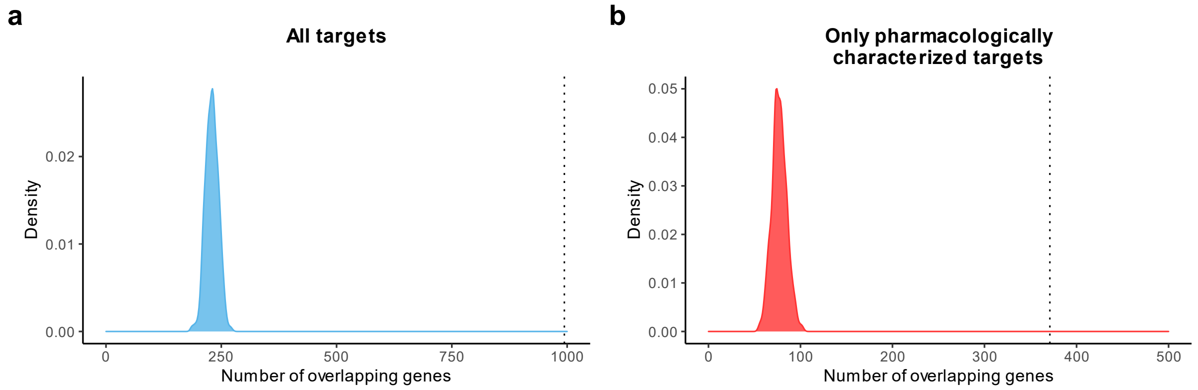

The overlap between drug targets and disease genes is four times higher than expected. a) Distribution of the number of overlapping genes between a set of random genes and the disease genes in our data set. The size of the random set equals the size of the drug direct targets. The black dotted line indicates the number of shared genes between our drug and disease sets which is significantly higher than expected (p-value < $2.2{\times}10^{-16}$). **b)** The same analysis for pharmacologically characterized drugs only (p-value < $2.2{\times}10^{-16}$).

Supplementary figure 3

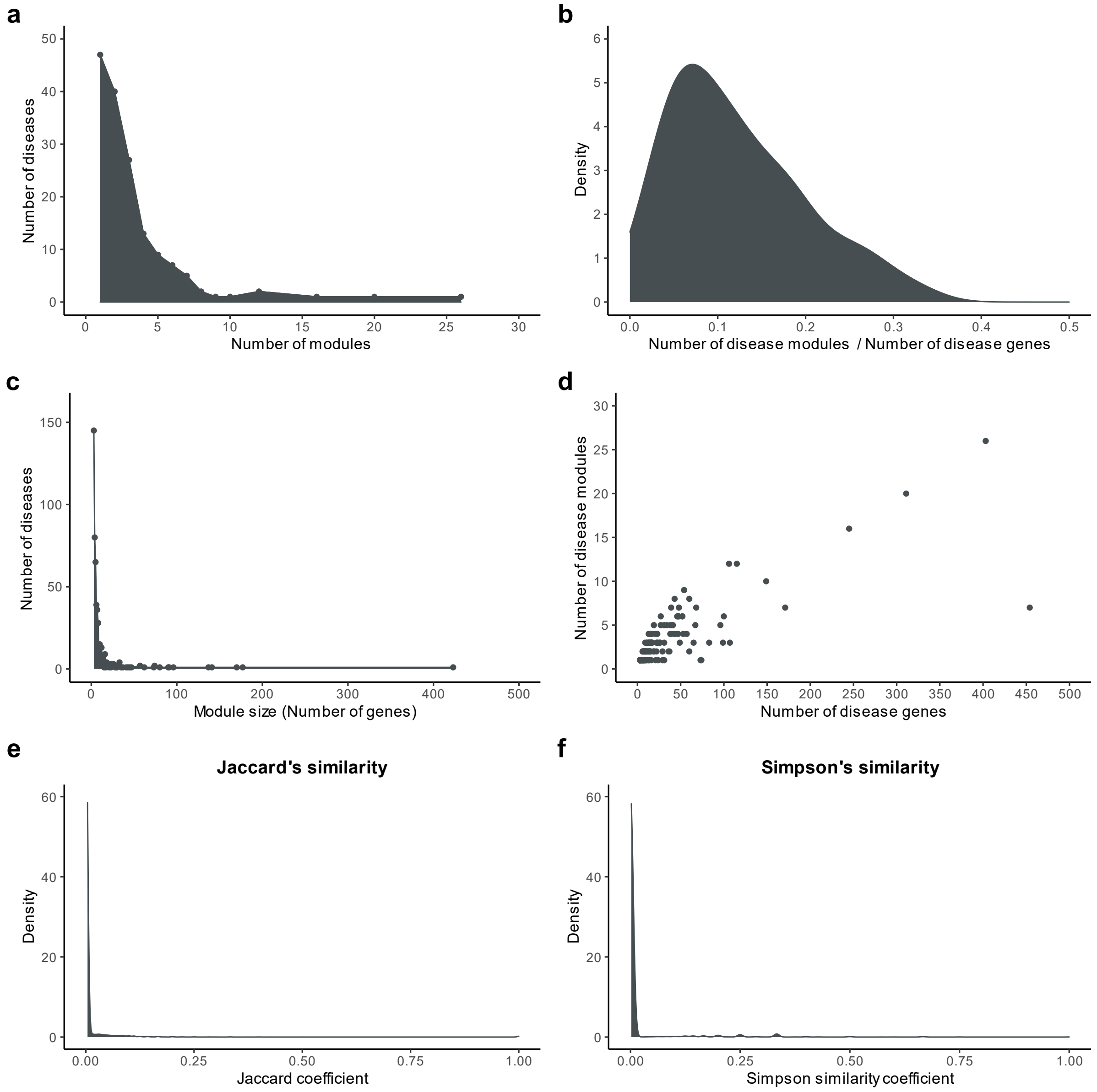

Characterization of disease modules. a) Distribution of the number of disease modules per disease. Only disease modules constituted by at least 3 genes are considered. **b)** Density plot of the normalized number of disease modules (number of modules for a disease/number of disease genes). **c)** Distribution of the size of disease modules, measured as number of genes in the module. Only modules with at least three genes are considered. **d)** Correlation between the number of disease genes and the number of modules found for each disease (rho = 0.775, p-value p-value < 2.2×10^{-16}). **e)** Distribution of the Jaccard similarity coefficients between all disease modules. **f)** Distribution of the Szymkiewicz–Simpson similarity coefficients between all disease modules.

Supplementary figure 4

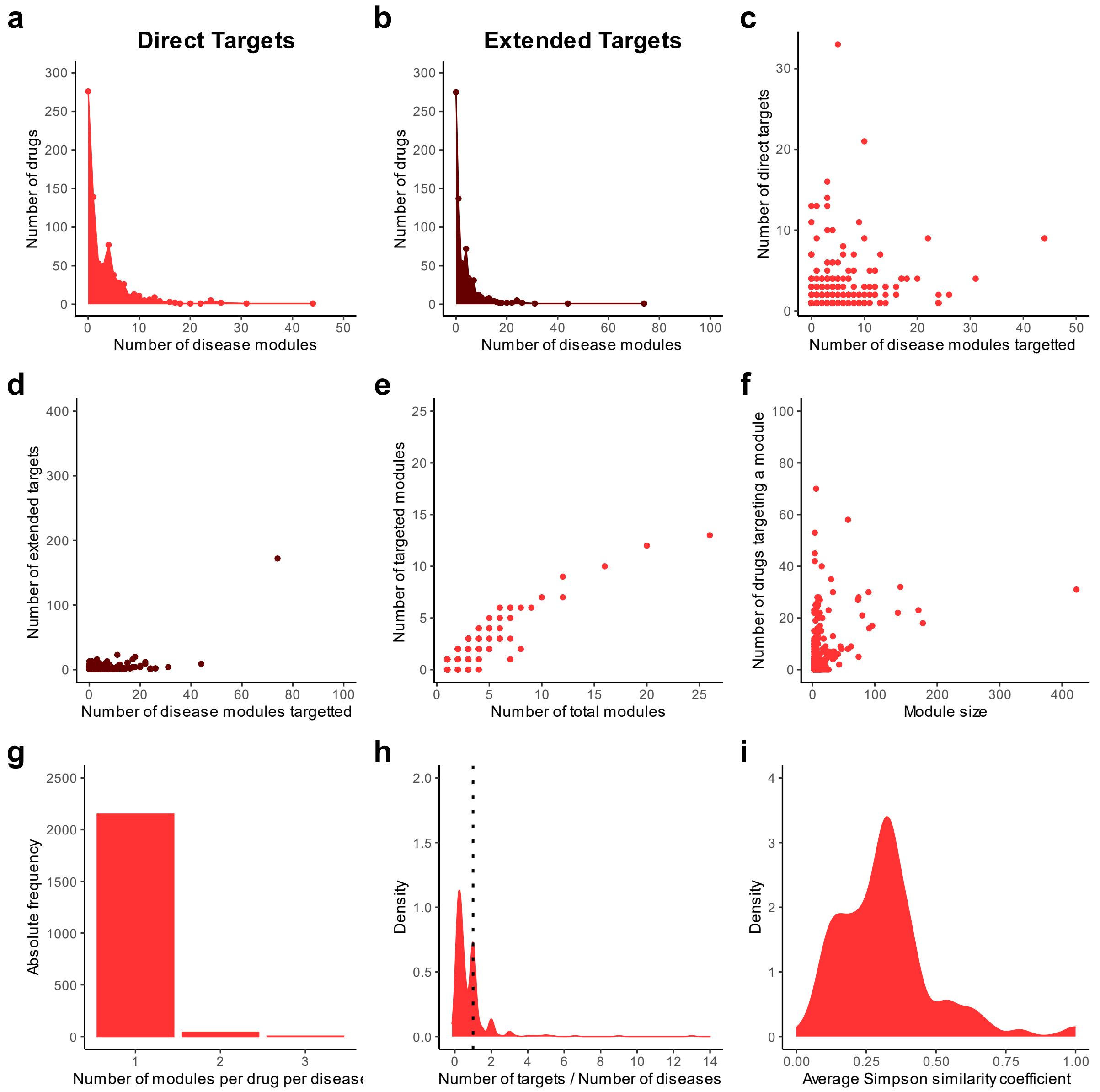

Most drugs target few disease modules. a) Distribution of the number of disease modules targeted per drug, considering the pharmacologically characterized drug direct targets. The number of disease modules targeted per drug ranges from 0 to 44, with a median of 1 and a standard deviation of 4.129. **b)** Distribution of the number of disease modules targeted per drug when extending the pharmacologically characterized direct targets. The distribution ranges from 0 to 230, with a median value of 1 and a standard deviation of 5.27. **c)** Correlation between the number of disease modules targeted by a drug and its number of pharmacologically characterized direct gene targets (rho = 0.220, p-value = 4.18×10^{-10}). **d)** Correlation between the number of disease modules targeted by a drug and its number of extended gene targets (rho = 0.245, p-value = 6.38×10^{-12}). **e)** Correlation between the total number of modules and the number of drug-targeted modules in a disease (rho = 0.759, p-value < 2.2×10^{-16}). **f)** Correlation between the size of a disease module and the number of drugs targeting it (rho = 0.347, p.value = 7.83×10^{-16}). **g)** Number of modules targeted per drug in a disease targeted by that drug. **h)** Barplot representing the absolute frequency of the number of different genes targeted by single drugs in different diseases. **i)** Density plot of the

Szymkiewicz–Simpson's similarity coefficient between disease modules targeted by single drugs (average Szymkiewicz–Simpson similarity coefficient = 0.317).

Supplementary figure 5

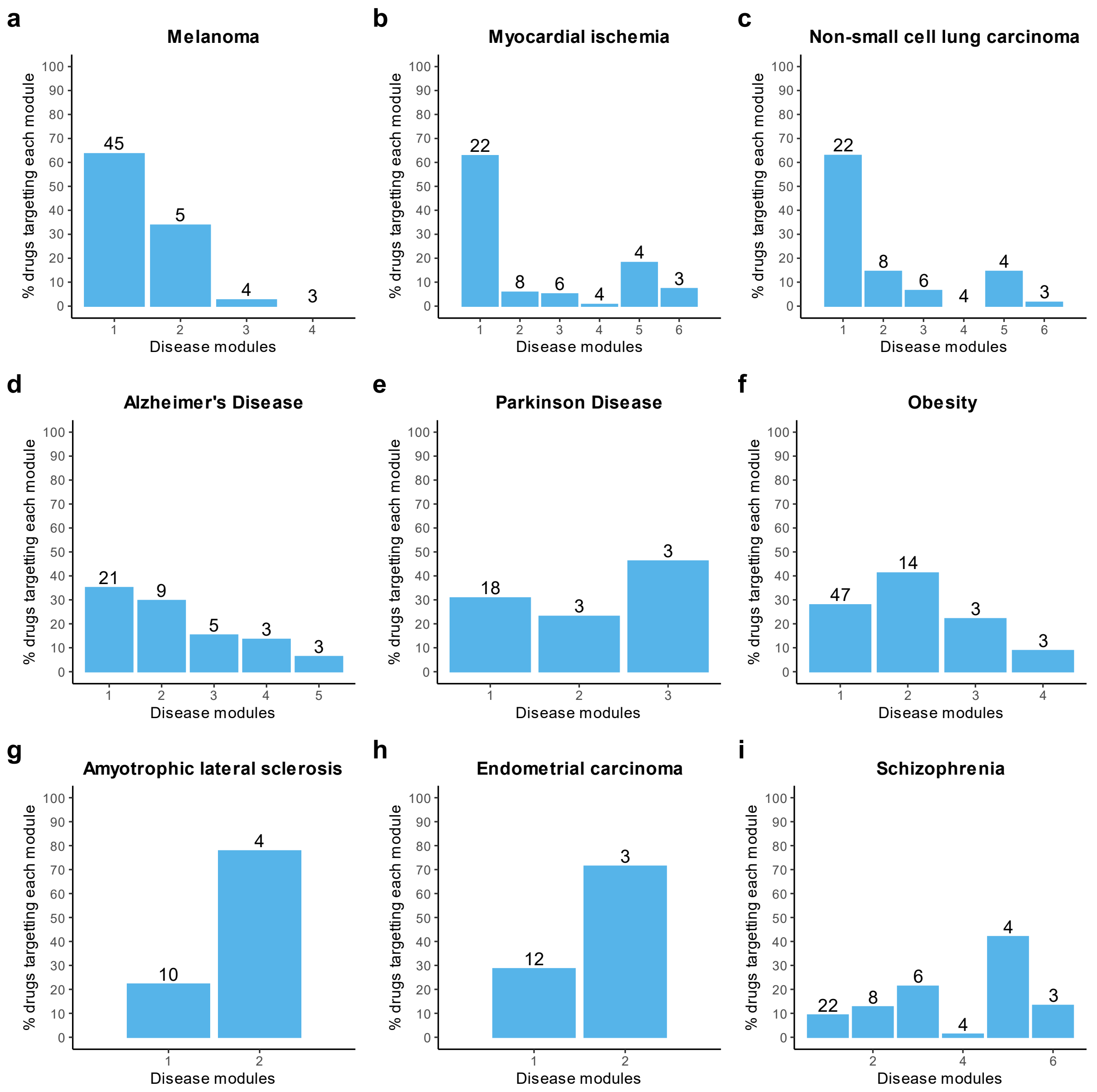

Examples of disease module targeting by drugs. In all panels the y-axis represents the percentage of drugs targeting each module, from all drugs targeting that disease. The x-axis represents the different modules per disease. **A)** *Melanoma*: approximately 60% of drugs targeting *melanoma* overlap with its biggest module, formed by 45 genes. **B)** *Myocardial ischemia:* 60% of drugs overlapping with the disease target the biggest module, composed of 80 genes. **C)** *Non-small cell lung carcinoma:* almost 60% of drugs overlap with a single module, composed of 28 genes. **D)** *Alzheimer's disease:* two modules of different sizes, 21 and 9 genes respectively, are each targeted by approximately 35% of drugs that overlap with *Alzheimer's disease*. **E)** *Parkinson's disease*: 80% of drugs covering the disease are distributed between two modules of 18 and 3 genes, respectively. **F)** *Obesity:* a single module made up by 14 genes is the most targeted one (by 40% of drugs overlapping with the disease), despite the presence of a bigger module constituted by 47 genes. **G)** *Amyotrophic lateral sclerosis:* the smallest module in the disease, made up by 4 genes, is the most targeted one. **H)** *Endometrial carcinoma:* the smallest module in the disease, with 3 genes, is the most targeted one. **I)**

Schizophrenia: despite the presence of a module with 22 genes, a single module with 4 genes is targeted by half of the drugs that overlap with the disease.

Supplementary tables

Supplementary table 1: Drug ranking based on the number of direct targets, extended targets, diseases with which the drug direct targets overlap, diseases with which the drug extended targets overlap, disease modules with which the drug direct targets overlap and disease modules with which the drug extended targets overlap.

Supplementary table 2: Drug ranking based on the number of pharmacologically characterized direct targets, extended targets when only the pharmacologically characterized direct targets are used as seeds for target set expansion, diseases with which the pharmacologically characterized drug direct targets overlap, diseases with which the drug extended targets overlap when using only pharmacologically characterized direct targets for the expansion, disease modules with which the drug pharmacologically characterized direct targets overlap and disease modules with which the drug extended targets overlap when only the pharmacologically characterized direct targets are used for the extension.

Supplementary table 3: "FDA interactions" drug ranking based on the number of drug indications.

Supplementary table 4: Drugs mapped to the COVID19 network.

Supplementary table 5: This table records, for each disease gene, the disease it is mapped to, the disease module it belongs to and the drugs that target it. A module number equal to 0 indicates that that gene does not belong to any module in that particular disease.

Supplementary table 6: Genes mapped to each disease module.
